# Biased stochastic motor dynamics drive bidirectional, processive translocation by the AAA+ disaggregase Hsp104

**DOI:** 10.64898/2026.08.09.743785

**Published:** 2026-08-10

**Authors:** JiaBei Lin, Jaskamaljot Kaur Banwait, Ayush Saurabh, Kim A. Sharp, Daniel R. Southworth, Steve Pressé, Aaron L. Lucius, Yale E. Goldman, James Shorter

## Abstract

Protein aggregation disrupts proteostasis and drives neurodegeneration. Hsp104 is a hexameric, ring-shaped AAA+ ATPase that dissolves protein aggregates, yet how hexamers translocate and extract polypeptides trapped in mechanically resistant aggregates remains unclear. Using substrates that recapitulate the physical constraints of aggregates, we establish that Hsp104 is a processive, bidirectional translocase that can dynamically switch direction while threading a single polypeptide. On mechanically restrained substrates and prions, Hsp104 hexamers execute biased stochastic transitions among three conformational states at individual interprotomer interfaces: closed, extended, and a previously unobserved hyperextended form. These transitions follow kinetically favored paths rather than a rigid rotary sequence. The resulting biased stochastic stepping, enabled by the conformational plasticity of Hsp104 hexamers, underpins operational adaptability and redefines the functional logic of AAA+ motors.

## Full Text Availability

The license terms selected by the author(s) for this preprint version do not permit archiving in PMC. The full text is available from the preprint server.

